# The associations of self-rated health with cardiovascular risk proteins: a proteomics approach

**DOI:** 10.1186/s12014-019-9258-9

**Published:** 2019-11-18

**Authors:** Xue Bao, Yan Borné, Songjiang Yin, Kaijun Niu, Marju Orho-Melander, Jan Nilsson, Olle Melander, Gunnar Engström

**Affiliations:** 1 Department of Cardiology, Nanjing Drum Tower Hospital, The Affiliated Hospital of Nanjing University Medical School, Nanjing, China; 2 Department of Clinical Sciences, Lund University, CRC 60:13, Jan Waldenströms gata 35, 20502 Malmö, Sweden; 3 Nutritional Epidemiology Institute and School of Public Health, Tianjin Medical University, Tianjin, China; 4 Department of Orthopedics, Jiangsu Province Hospital of Chinese Medicine, Nanjing University of Chinese Medicine, Nanjing, China

**Keywords:** C–C motif chemokine 20, Leptin, Proteomic, Self-rated health

## Abstract

**Background:**

Though subjective, poor self-rated health (SRH) has consistently been shown to predict cardiovascular disease (CVD). The underlying mechanism is unclear. This study evaluates the associations of SRH with biomarkers for CVD, aiming to explore potential pathways between poor SRH and CVD.

**Methods:**

Based on the Malmö Diet and Cancer Cardiovascular Cohort study, a targeted proteomics approach was used to assess the associations of SRH with 88 cardiovascular risk proteins, measured in plasma from 4521 participants without CVD. The false discovery rate (FDR) was controlled using the Benjamini and Hochberg method. Covariates taken into consideration were age, sex, traditional CVD risk factors (low-density lipoprotein cholesterol, systolic blood pressure, anti-hypertensive medication, diabetes, body mass index, smoking), comorbidity, life-style and psycho-social factors (education level, living alone, alcohol consumption, low physical activity, psychiatric medication, sleep duration, and unemployment).

**Results:**

Age and sex-adjusted associations with SRH was found for 34 plasma proteins. Nine of them remained significant after adjustments for traditional CVD risk factors. After further adjustment for comorbidity, life-style and psycho-social factors, only leptin (β = − 0.035, corrected *p *= 0.016) and C–C motif chemokine 20 (CCL20; β = − 0.054, corrected *p *= 0.016) were significantly associated with SRH.

**Conclusions:**

Poor SRH was associated with raised concentrations of many plasma proteins. However, the relationships were largely attenuated by adjustments for CVD risk factors, comorbidity and psycho-social factors. Leptin and CCL20 were associated with poor SRH in the present study and could potentially be involved in the SRH–CVD link.

## Background

How people rate their own health is a subjective but surprisingly sensitive and reliable assay for evaluation of general well-being. A meta-analysis of 22 prospective, community-based cohort studies has reported that compared to participants with “excellent” self-rated health (SRH), mortality risk among those who rated their health as “poor” has almost doubled even after adjustment for co-morbidity, depression, and cognitive and functional status [1]. The association of poor SRH with mortality is at least partly driven by its association with cardiovascular diseases (CVDs). This hypothesis is supported by a meta-analysis demonstrating a strong association between poor SRH and incidence of cardiovascular mortality in populations with or without previous CVD [2]. For people without prior CVD, a significant predictive value of poor SRH for onset of CVD events has also been observed by several cohort studies [3–9].

The mechanisms underlying the association between poor SRH and CVD remain unclear. SRH is a comprehensive indicator of health status that closely associates with material, behavioral, personality and psycho-social factors [10, 11]. These socioeconomic inequalities affect individual’s healthcare resource use and predisposition to better or worse health, including CVD risk and mortality [12–15], which could be one important reason behind the association between poor SRH and CVD. SRH may also influence immune responses or autonomic nervous system [16–19], or reflect to some extent the functional impairment, the physical morbidity [3, 20, 21], and the objective measures of cardiovascular risk [2, 3, 7, 9]. For people with poor SRH, certain circulating inflammatory markers have been observed to be elevated, including the more commonly studied interleukin (IL)-6 and C-reactive protein [17, 22–25] and others (IL-1β, IL-1rα, erythrocyte sedimentation rate, tumor necrosis factor-α, etc.) [16, 19, 26].

In this study, we aimed to use a recently-developed targeted proteomics approach to explore the change in cardiovascular proteomics associated with SRH. Being highly sensitive and specific, the implement of this proteomics methodology may provide a novel insight into biologically plausible mechanistic pathways between poor SRH and CVD.

## Materials and methods

### Participants

**Fig. 1:**
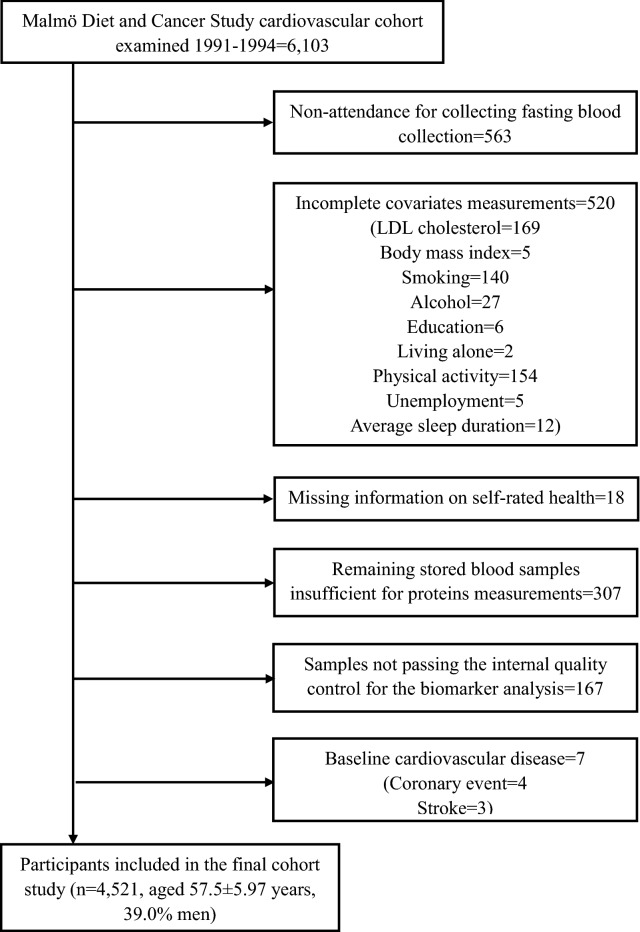
Study population flow chart (n = 4521)

The Malmö Diet and Cancer (MDC) study is a large prospective cohort study among residents living in the Swedish town of Malmö [27]. Between 1991 to 1994, 6103 randomly selected participants from the MDC study were invited to participate in the Malmö Diet and Cancer Cardiovascular Cohort (MDC-CV) study aiming at investigating the epidemiology of carotid artery atherosclerosis [28]. Among them, 5540 participants undertook a second visit for collecting fasting plasma samples, of which 5002 had complete data on covariates. Out of these, 307 subjects with insufficient plasma stored for assessing proteins and 167 subjects who did not pass the internal quality control for the protein analyses were excluded. Of the remaining 4528 participants, we further excluded those with prevalent CVDs (n = 7), leading a final sample size of 4521 for cohort analysis (1763 men and 2758 women. mean age, 57.5 ± 5.97 years) (Fig. 1).

Written informed consent have been obtained from all participants. The study has been approved by the Regional Ethical Review Board in Lund, Sweden (LU 51/90) and was carried out in accordance with the Helsinki Declaration.

### Self-rated health

Self-rated health was assessed on an ordinal scale ranging from 1 to 7, based on subjects’ answers to a questionnaire item: “How do you feel right now, physically and mentally, with respect to your health and well-being?”. While “1” indicates “feel very bad, could not feel worse”, “7” indicates “feel very well, could not feel better”.

### Proteomic analysis

Fasting EDTA-plasma samples were stored at − 80 °C immediately after collection until protein analysis. Ninety-two CVD-related proteins were simultaneously measured by the SciLifeLab analysis service (Uppsala, Sweden) using Proseek^®^ Multiplex CVD I^96×96^ reagent kit [29]. The reagents are based on proximity extension assay (PEA) technology, where 92 pairs of oligonucleotide-labeled antibody probes were used to detect the corresponding target proteins in a homogeneous assay [30, 31]. Only correctly matched probe pairs will generate detectable and quantifiable signals for a Fluidigm^®^ Biomark™ HD real-time PCR platform, making the technology of significantly higher specificity and sensitivity than traditional multiplex immunoassays [29–31]. Quantitative PCR quantification cycles (Cq) corrected for technical variation by the Inter-plate Control (IPC) generate “Normalized Protein Expression (NPX)” values, which are arbitrary units on log2 scale. A higher NPX value corresponds to a higher protein level. Samples deviate less than ± 0.3 from the median value for the incubation and detection controls will pass quality control [32]. The mean intra-assay (within-run) and inter-assay (between-run) coefficient of variations were 8% (range 4–13%) and 15% (range 11–39%), respectively. Detailed introduction of cardiovascular proteomic panel, PEA technology, assay performance, quality control and validation is available on the Olink webpage (http://www.olink.com). Four proteins had a call rate < 75%: extracellular newly identified RAGE-binding (EN-RAGE, n = 101), beta-nerve growth factor (Beta-NGF, n = 451), IL-4 (n = 12), and B-type natriuretic peptide (BNP, n = 636) were removed from main analyses (none of them were significantly associated with SRH, data not shown). This resulted in 88 proteins for final analyses. Proteins with levels below the lower limit of detection (LOD) were considered to have a value of LOD/2.

### Other measurements and definitions

Information on alcohol consumption and smoking, medication (anti-hypertensive and psychiatric medication), comorbidity (i.e. ventricular ulcer, cancer, asthma/chronic bronchitis, rheumatoid arthritis, inflammatory bowel disease, and kidney stone), physical activity, education level, living alone, average sleep duration, and unemployment were obtained from a health questionnaire completed by the participants and their 7-day personal diary. Smoking was treated as a two-category variable: smokers or non-smokers. Men with alcohol intake > 40 g/day or women with alcohol intake > 30 g/day were considered to have high alcohol consumption. Use of anti-hypertensive and psychiatric drug was identified based on the drugs listed by participants. Anatomical Therapeutic Chemical Classification codes N05 and N06 were used for psychiatric drugs and C02, C03, C07 and C08 were used for anti-hypertensive drugs. Subjects with self-reported diabetes or diabetes treatment or with a fasting venous whole blood glucose higher than 6.1 mmol/L (corresponding to 7.0 mmol/L when using fasting plasma glucose to diagnose diabetes [33]) were considered to have diabetes. An overall leisure-time physical activity score was calculated by multiplying an activity-specific intensity coefficient and the corresponding duration [34]. People in the lowest quartile of physical activity score were considered to have low physical activity. People with a minimum of a university degree were considered to have a high educational level. They also reported whether they were living alone and whether they were unemployed. A weighted average sleep duration was calculated for individuals based on average sleep duration (hours) on weekdays and weekends [(weekday × 5) + (weekend × 2)/7], and then categorized as (≤ 6, 6–8, ≥ 8 h). Body mass index (BMI) was calculated as weight in kilograms divided by the square of height in meters (kg/m^2^). Blood pressure (mmHg) was measured after the participants had rested for 10 min in a supine position. Blood samples were drawn after an overnight fast. LDL concentration (mmol/L) was estimated using Friedewald’s formula [35].

Incidence of CVD and mortality up to December 31st, 2016, was monitored by data linkage with the Swedish in-patient register and the cause of death register. Incident CVD included new cases of coronary event (fatal or non-fatal myocardial infarction or death due to ischemic heart disease) or stroke diagnosed according to the International Classification of Diseases 9th or 10th revision [36].

### Statistical analysis

Protein values were Z-score standardized in all analyses. Pearson’s partial correlation tests were conducted between each pair of proteins after adjusting for age and sex. Baseline characteristics for subjects included in this study (n = 4521) are demonstrated for those with SRH higher or lower than the median level (SRH = 5). Continuous variables are all normally distributed and thus presented as mean ± SD, while categorical variables are presented as percentages. Comparisons between the two groups were performed using univariate Chi-square or student t-tests. The hazard ratio of SRH for incident CVD (or mortality) was calculated using Cox proportional hazard regression models, with time-scale defined as time to follow-up until incident CVD (or mortality), emigration, death or end of follow-up (2016-12-31). Since only few participants scored their health as 1 or 2 (n = 46 and 71, respectively), participants with an SRH value of 1–3 were combined into one group.

Linear regression models were separately conducted for every single protein to investigate its association with SRH (as the independent variable). In primary analyses, only age and sex were adjusted for and forest plot was used to visualize the associations. In a second model, traditional cardiovascular risk factors were included (age, sex, smoking, LDL-cholesterol, diabetes, BMI, systolic blood pressure and anti-hypertensive treatment). Finally, comorbidity, life-style and psycho-social risk factors were added to the model (comorbidity, alcohol consumption, education level, living alone, low physical activity, psychiatric medication, average sleep duration, and unemployment). The covariates were examined for multi-collinearity using the Variance Inflation Factor (VIF) and it was found that VIF was < 2 for all independent variables. Since multiple testing was involved in both analyses, *p* values were corrected for false discovery rate using the Benjamini and Hochberg method [37]. For proteins of significant associations with SRH after correction, possible effect modifications by covariates were explored by introducing an interaction term in the multivariate model (one term per covariate at a time). A two-tailed *p* value of < 0.05 was considered as statistically significant. All analyses were performed using the Statistical Analysis System version 9.3 for Windows (SAS Institute Inc., Cary, NC, USA).

## Results

### Study population characteristics

The baseline characteristics of the study population according to their SRH values are shown in Table 1. As compared to participants with lower SRH (median: 5), those with higher SRH were more likely to be older, or have lower BMI or systolic blood pressure. A greater proportion of them were males and non-smokers. They also tended to be more physically active and less likely to live alone, take anti-hypertensive or psychiatric medication, or have diabetes or other comorbidities. A greater proportion of them slept 6–8 h per day, and a smaller proportion of them slept less than 6 h per day.

**Table 1:** Participants’ characteristics according to their self-rated health (n = 4521)

	Self-rated health		*p*^a^
	Lower than or equal to 5 (n = 2356)	Higher than 5 (n = 2165)	
Age (years)	57.3 (± 5.86)	57.7 (± 6.08)	0.04
Sex (male, %)	875 (37.1%)	888 (41.0%)	< 0.01
Body mass index (kg/m^2^)	25.9 (± 4.17)	25.2 (± 3.57)	< 0.0001
Systolic blood pressure (mmHg)	141.7 (± 19.8)	139.7 (± 17.7)	< 0.001
Low-density lipoprotein cholesterol (mmol/L)	4.17 (± 0.99)	4.16 (± 0.96)	0.78
Smoker (%)	670 (28.4%)	498 (23.0%)	< 0.0001
Anti-hypertensive medication (%)	439 (18.6%)	235 (10.9%)	< 0.0001
High alcohol consumption (%)	94 (3.99%)	64 (2.96%)	0.06
High education (%)	646 (27.4%)	615 (28.4%)	0.46
Living alone (%)	579 (24.6%)	462 (21.3%)	< 0.01
Low physical activity (%)	603 (25.6%)	466 (21.5%)	< 0.01
Diabetes (%)	212 (9.00%)	130 (6.00%)	< 0.001
Comorbidity (%)^b^	841 (35.7%)	517 (23.9%)	< 0.0001
Sleep duration per day (%)			
≤ 6 h	332 (14.1%)	181 (8.36%)	< 0.0001
6–8 h	1250 (53.1%)	1233 (57.0%)	< 0.01
≥ 8 h	774 (32.9%)	751 (34.7%)	0.19
Unemployment (%)	111 (4.71%)	86 (3.97%)	0.22
Psychiatric medication (%)	163 (6.92%)	42 (1.94%)	< 0.0001

Values expressed are means (± standard deviation) or percentages

^a^Analysis of variance or logistic regression analysis

^b^Comorbidity included ventricular ulcer, cancer, asthma/chronic bronchitis, rheumatoid arthritis, inflammatory bowel disease, and kidney stone

### The predictive value of poor SRH for adverse outcomes

During the follow-up, 1445 deaths and 830 CVD events were recorded. Worse SRH was associated with a graded increase in risk across all outcomes, which persisted after sequential adjustment. In models adjusted for age, sex, traditional CVD risk factors, comorbidity, life-style and psycho-social factors, participants in the poor SRH scores had a markedly elevated risk of CVD (HR, 1.64; 95% CI 1.25–2.15; *p* for trend < 0.0001) and a slightly elevated risk of mortality (HR, 1.20; 95% CI 0.97–1.47; *p* for trend = 0.02) compared with those with a SRH score of 7. In total, results from the Cox regression analyses (Table 2) supported a predictive value of poor SRH for adverse outcomes (i.e. CVD and mortality).

**Table 2:** Incidence of cardiovascular disease (CVD) and mortality in relation to self-rated health

	Self-rated health					*p* for trend^a^
	7	6	5	4	1–3	
N (= 4521)	904	1261	1184	772	400	–
Incident CVD (n = 830)	145	204	230	158	93	–
Incidence (per 1000 person-years)	8	8	10	10	12	–
Model 1^b^	Reference	1.06 (0.85, 1.31)	1.38 (1.12, 1.70)	1.55 (1.23, 1.94)	2.00 (1.54, 2.60)	< 0.0001
Model 2^c^	Reference	1.09 (0.88, 1.35)	1.32 (1.07, 1.63)	1.43 (1.14, 1.79)	1.70 (1.30, 2.21)	< 0.001
Model 3^d^	Reference	1.13 (0.91, 1.40)	1.35 (1.10, 1.67)	1.41 (1.12, 1.77)	1.64 (1.25, 2.15)	< 0.0001
Incident mortality (n = 1445)	289	351	387	269	149	–
Incidence (per 1000 person-years)	15	13	15	16	18	–
Model 1^b^	Reference	0.95 (0.81, 1.11)	1.17 (1.01, 1.36)	1.32 (1.12, 1.56)	1.65 (1.36, 2.02)	< 0.0001
Model 2^c^	Reference	0.97 (0.83, 1.13)	1.15 (0.98, 1.34)	1.23 (1.04, 1.45)	1.36 (1.11, 1.67)	< 0.0001
Model 3^d^	Reference	0.98 (0.84, 1.15)	1.12 (0.96, 1.31)	1.14 (0.96, 1.35)	1.20 (0.97, 1.47)	0.02

*HR* hazard ratio

^a^Analysis by Cox proportional hazards model

^b^Adjusted for sex and age

^c^Adjusted for sex, age, body mass index, smoking, low-density lipoprotein cholesterol, systolic blood pressure, anti-hypertensive drug medication, and diabetes

^d^Adjusted for sex, age, body mass index, alcohol consumption, smoking, low-density lipoprotein cholesterol, systolic blood pressure, anti-hypertensive drug medication, diabetes, comorbidity, education level, living alone, low physical activity, sleep duration, unemployment, and psychiatric medication

### Plasma proteins in relation to SRH

**Fig. 2:**
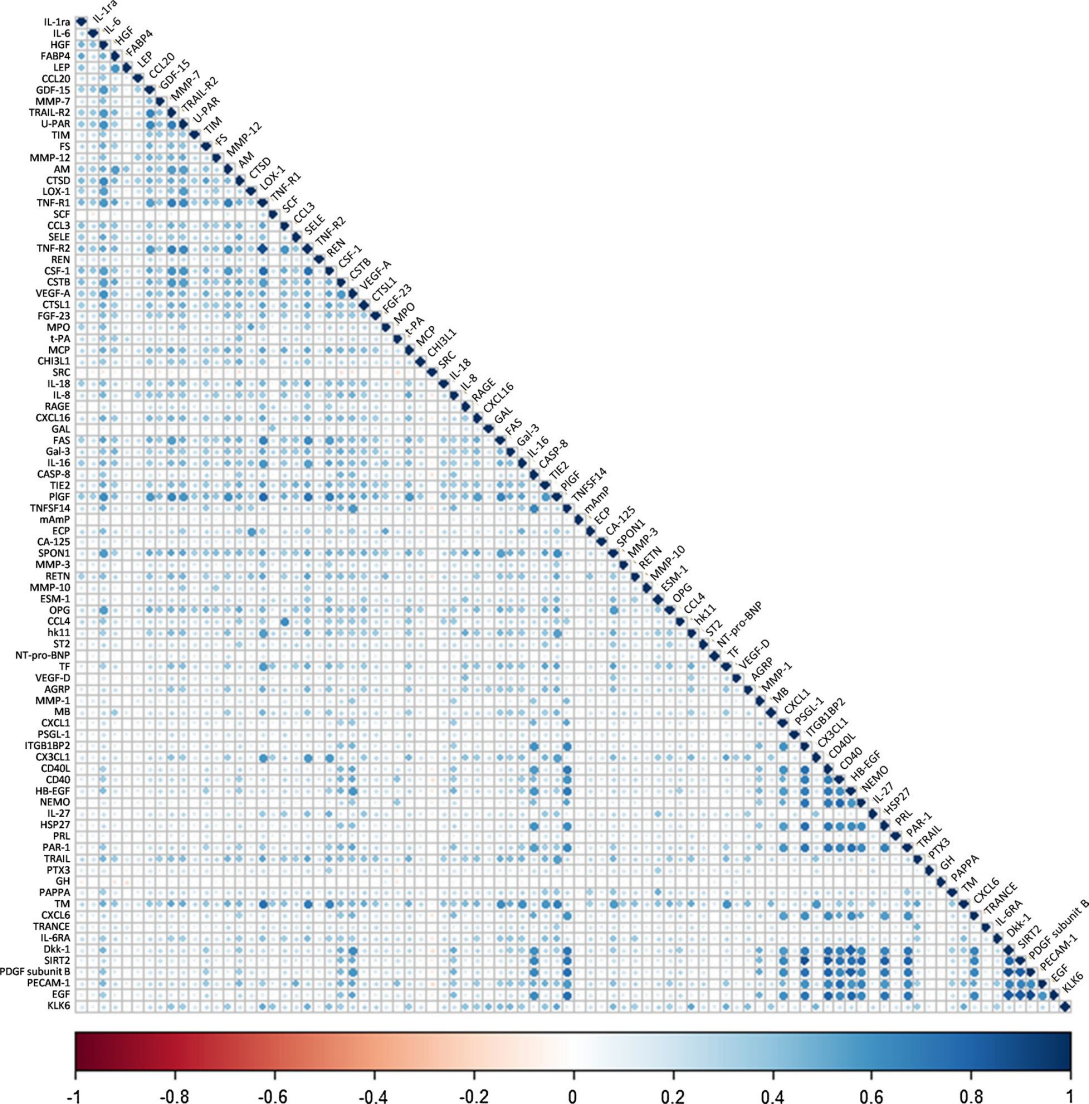
Correlation matrix of the 88 measured proteins. Correlation coefficients were calculated between every two proteins using Pearson’s partial correlation tests, adjusted for age and sex. Higher positive (negative) correlation corresponding to darker blue (red)

**Fig. 3:**
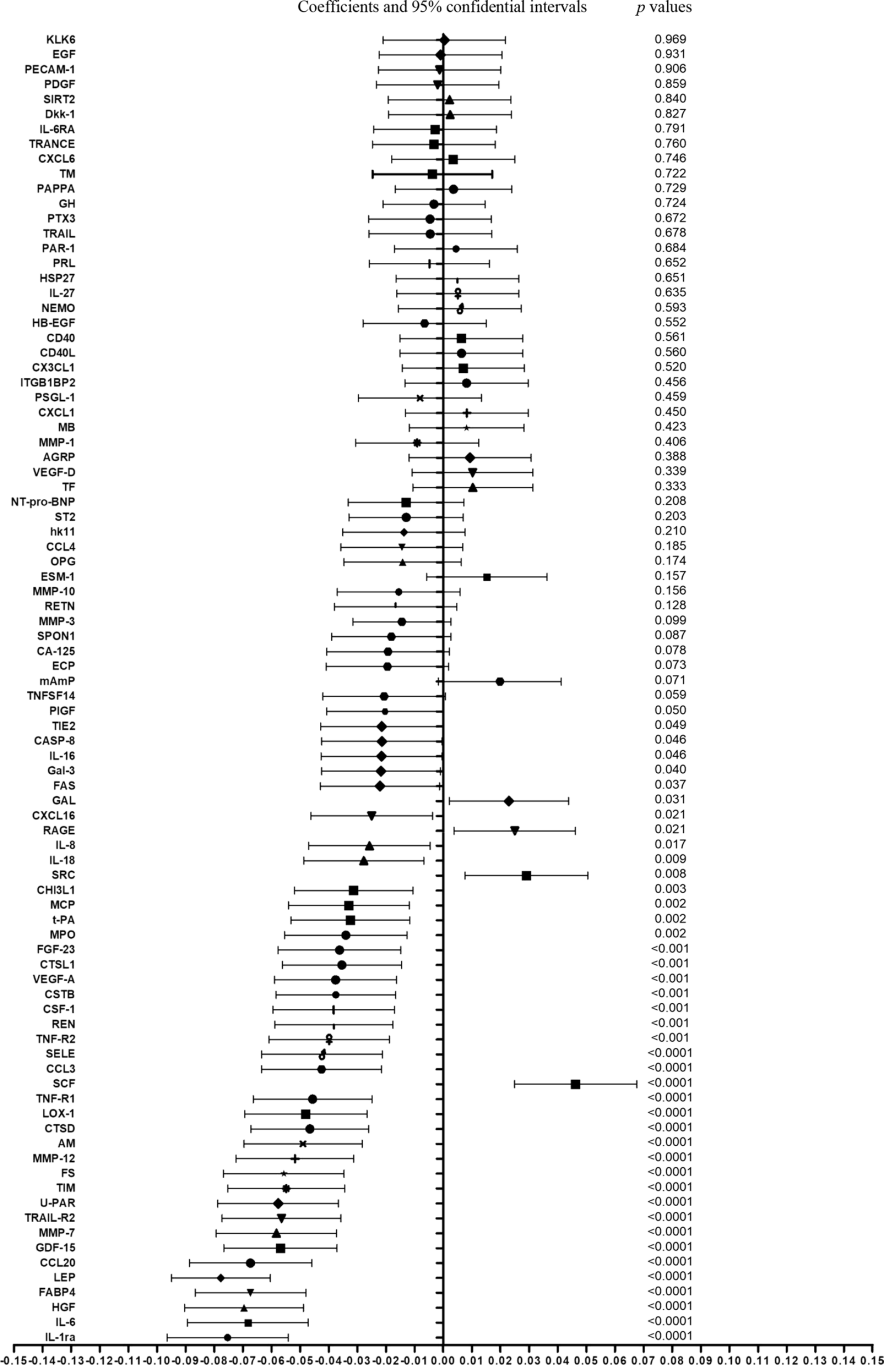
Forest plot of the associations between self-rated health (independent variable) and the 88 proteins (dependent variables). Coefficients, 95% confidential intervals, and *p* values were obtained from linear regression models conducted separately for each protein, adjusted for age and sex

As depicted in Fig. 2, high correlations can be observed across pairs of proteins. After adjusting for age and sex, nominal significant associations (*p *< 0.05) were found of SRH with 42 of the 88 proteins examined (Fig. 3). Thirty-four of them remained significant after correcting for multiple testing (FDR < 5%).

After further adjustment for traditional cardiovascular risk factors (smoking, BMI, LDL, diabetes, systolic blood pressure and antihypertensive treatment) nine proteins remained significant after correcting for multiple testing (leptin, CCL20, IL-1 receptor antagonist protein, IL-6, matrix metalloproteinase-7, lectin-like oxidized LDL receptor 1, urokinase plasminogen activator surface receptor, matrix metalloproteinase-12, follistatin; corrected *p *< 0.001, < 0.001, = 0.011, = 0.015, = 0.021, = 0.034, = 0.034, = 0.034, = 0.034, respectively). When comorbidity and psycho-social factors were added to the model, eight plasma proteins were related to SRH with nominal significance; two of them (leptin, β = − 0.035, corrected *p *= 0.016 and CCL20, β = − 0.054, corrected *p *= 0.016) were significant after correction for multiple testing; Table 3).

**Table 3:** Associations of self-rated health with leptin or C–C motif chemokine 20 in whole sample and sub-groups

	Standardized estimate	Standardized error	*p*
Leptin			
Whole sample (n = 4521)	− 0.035	0.007	0.016^a^
Sex			
Males (n = 1763)	− 0.055	0.012	0.003
Females (n = 2758)	− 0.036	0.009	0.015
Body mass index			
< 25 (n = 2223)	− 0.038	0.013	0.020
≥ 25 (n = 2298)	− 0.071	0.010	< 0.0001
High alcohol consumption			
No (n = 4363)	− 0.031	0.007	< 0.01
Yes (n = 158)	− 0.144	0.041	0.012
Systolic blood pressure			
< 140 (n = 2080)	− 0.008	0.014	< 0.0001
≥ 140 (n = 2441)	− 0.073	0.012	< 0.0001
C–C motif chemokine 20			
Whole sample (n = 4521)	− 0.054	0.011	0.016^a^
Smoking status			
Non-smokers (n = 3353)	− 0.079	0.013	< 0.001
Smokers (n = 1168)	0.016	0.022	0.598

Multiple linear regression with leptin or C–C motif chemokine 20 as dependent variable, self-rated health as independent variable, and sex, age, body mass index, alcohol consumption, smoking, low-density lipoprotein cholesterol, systolic blood pressure, anti-hypertensive drug medication, diabetes, comorbidity, education level, living alone, low physical activity, sleep duration, unemployment, and psychiatric medication as covariates

^a^*p* values corrected for false discovery rate

Interaction tests showed that the adjusted association between leptin and SRH was modified by sex, BMI, alcohol consumption, and systolic blood pressure (interaction *p *= 0.019, < 0.001, = 0.033, = 0.035, respectively), while the association between CCL20 and SRH was modified by smoking (interaction *p *= 0.006). Results of the corresponding subgroup analyses are presented in Table 3. The association of SRH with leptin was relatively strong among males (n = 1763), or people with obesity (n = 2298) or high alcohol consumption (n = 158) or elevated systolic blood pressure (n = 2441). The association between SRH and CCL20 was significant among non-smokers (n = 3353) but not smokers. The association between SRH and mortality and CVD, respectively, was only marginally changed after additional adjustment for leptin and CCL20 in multivariate Cox regression models. The hazard ratios of SRH 1–3 versus 7 for CVD and mortality decreased from 1.64 (1.25, 2.15) to 1.62 (1.23, 2.13), and from 1.20 (0.97, 1.47) to 1.18 (0.96, 1.46), respectively (data not shown).

## Discussion

Many epidemiological studies reported that poor SRH is a strong predictor for subsequent mortality, even after extensive adjustments for other potential risk factors [1–9]. Consistent with these previous observations, our results showed that the participants in the poor SRH scores had a markedly elevated risk of CVD and mortality events. The underlying biological cause for this relationship is unclear. In this study, we extend existing literature on the SRH–CVD link by applying a proteomic analysis. Poor SRH was associated with raised concentrations of many plasma proteins after adjustments for age and sex. After adjustments for traditional cardiovascular risk factors, nine proteins were still significantly associated with SRH. The relationships were largely attenuated by further adjustments for comorbidity and psycho-social factors. For two proteins, leptin and CCL20, we found significant relationships even after adjustments for multiple risk factors, and these proteins could potentially have a role in the SRH–CVD link beyond traditional risk factors.

Self-rated health is a summative measure of a person’s overall assessment of health status. As such, many psychological as well as medical factors could affect SRH. It is noteworthy that some variables, which usually are related to poor health, were unrelated to SRH in this study (e.g. LDL-cholesterol, education, and unemployment), while the relationships with poor SRH were strong for e.g. smoking, diabetes and anti-hypertensive medication (Table 1). It is not possible to make any conclusions of the causal relationships between SRH and the various plasma proteins. However, we conclude that poor SRH was significantly associated with many of the plasma proteins in this targeted CVD panel. We also conclude that traditional CVD risk factors, as well as factors related to comorbidity, life-style or psycho-social factors largely account for the relationships between SRH and plasma proteins.

Inflammation has been firmly established as crucial to the development of CVD [38]. The activation of local arterial inflammation or system immune responses could together lead to initiation or progression of atherosclerotic plaques, and even complicated atherosclerotic lesions. Meanwhile, poor SRH could be linked to immune responses through the elevation of circulating inflammatory and immune cytokines [16, 17, 19, 22–26]. These facts raised a possibility that immune responses may be the underlying mechanism to explain the relationship between poor SRH and CVD. In the current study, using a targeted proteomics approach, 34 proteins were found to be significantly associated with SRH after adjusting by age and sex. Noteworthily, many of these proteins were related to immune or inflammatory responses.

After multivariate adjustment, an association between poor SRH and CCL20 was still observed in our study. CCL20 is a recently discovered CC chemokine which functions together with its selective receptor CCR6 to mediate the chemoattraction of immature dendritic cells and effector and memory T- and B-cells [39]. Whereas this association has not yet been reported before, a pivotal role of T helper 17 (Th17) cells in the pathophysiology of depression have already been demonstrated in recent studies [40, 41]. CCL20 can be produced by Th17 [42] and is important for Th17 cell migration and tissue inflammation [43]. The CCL20–CCR6 axis in epithelial cells of choroid plexus has been proposed as a key point for Th17 cells to enter the central nervous system, which may further trigger local inflammation [44, 45] and therefore might potentially contribute to poor SRH.

The coexistence of increased circulating leptin and depression has been previously demonstrated, though in studies with small sample sizes and clinical heterogeneity [46, 47]. In addition, only two studies [48, 49] have investigated the association between SRH and leptin. In this prospective study, after multivariate adjustments, the association of poor SRH with higher leptin was significant in both sexes and was relatively stronger in men (interaction *p *= 0.019). Leptin is an adipose-derived hormone that can control body weight by inhibiting appetite and increasing energy expenditure [50]. However, the anti-obesity role of leptin is usually thwarted by leptin resistance, which leads to elevated leptin levels in obesity [51]. Leptin resistance has also been proposed as a potential interface of inflammation and metabolic disturbance linking obesity and CVD [52]. In the present study, a much stronger association between poor SRH and high leptin was observed in participants with BMI higher vs. lower than 25 kg/m^2^ (interaction *p *< 0.001). It is therefore speculated that for people with poor SRH, obesity accompanied by elevated leptin concentration and leptin resistance may contribute to some extent the subsequent cardiovascular risk [53]. As an obesity-related disorder, leptin resistance may be linked with mood status via several biological pathways [54–56]. In adult mice, targeted deletion of leptin receptors in the hippocampus and cortex leads to hyperleptinemia [55] and depression-related behaviors [54]. Leptin receptor deficiency is also associated with resistance to anti-depressive medications [55]. Thus, a link among elevated leptin, poor SRH, and subsequent risk for CVD could be explained.

Strengths of this study included a large sample size and the application of a highly sensitive and specific proteomics approach. However, the reliability of our results is limited by lack of replication samples. The 88 proteins measured in this study only constitute a minor subpopulation of the CVD-related proteins, and their associations with SRH were only cross-sectionally investigated. Since SRH appears to be a summative assessment of various aspects of health, we cannot rule out residual confounding from some aspects that can hardly be measured, or reverse causality. Therefore, the exact biological mechanisms underlying the association between SRH and cardiovascular outcomes cannot be illustrated in the present study. Nevertheless, we supported a biological change associated with subjective measurement, which helps to explain the predictive value of SRH for future outcomes.

## Conclusions

Poor SRH was associated with raised concentrations of many plasma proteins. However, the relationships were largely attenuated by adjustments for traditional CVD risk factors and factors related to comorbidity, life-style and psycho-social factors. Leptin and CCL20 were associated with poor SRH after multiple adjustments and could potentially be involved in the SRH–CVD link.

## Data Availability

The datasets used during the current study belongs to Lund University and applications for studies using the MDC cohort can be addressed to the MKC steering committee. Email: Anders.Dahlin@med.lu.se.

## Abbreviations

BMI: body mass index
Cq: quantification cycle
CVD: cardiovascular disease
FDR: false discovery rate
LOD: lower limit of detection
MDC: Malmö Diet and Cancer
MDC-CV: Malmö Diet and Cancer Cardiovascular Cohort
NPX: Normalized Protein Expression
PEA: proximity extension assay
SRH: self-rated health
Th17: T helper 17
VIF: Variance Inflation Factor
AGRP: agouti-related protein
AM: adrenomedullin
Beta-NGF: beta-nerve growth factor
BNP: natriuretic peptides B
CA-125: ovarian cancer-related tumor marker CA 125
CASP-8: caspase-8
CCL20: C–C motif chemokine 20
CCL3: C–C motif chemokine 3
CCL4: C–C motif chemokine 4
CD40: CD40L receptor
CD40L: CD40 ligand
CHI3L1: chitinase-3-like protein 1
CSF1: macrophage colony-stimulating factor 1
CSTB: cystatin-B
CTSD: cathepsin D
CTSL1: cathepsin L1
CX3CL1: fractalkine
CXCL1: C-X-C motif chemokine 1
CXCL16: C-X-C motif chemokine 16
CXCL6: C-X-C motif chemokine 6
DKK-1: dickkopf-related protein 1
ECP: eosinophil cationic protein
EGF: epidermal growth factor
EN-RAGE: protein S100-A12
ESM-1: endothelial cell-specific molecule 1
FABP4: fatty acid-binding protein, adipocyte
FAS: tumor necrosis factor receptor superfamily member 6
FGF-23: fibroblast growth factor 23
FS: follistatin
GAL: galanin peptides
Gal-3: galectin-3
GDF-15: growth/differentiation factor 15
GH: growth hormone
HB-EGF: heparin-binding EGF-like growth factor
HGF: hepatocyte growth factor
hK11: kallikrein-11
HSP27: heat shock 27 kDa protein
IL-16: interleukin-16
IL-18: interleukin-18
IL-1RA: interleukin-1 receptor antagonist protein
IL-4: interleukin-4
IL-6: interleukin-6
IL-6RA: interleukin-6 receptor subunit alpha
IL-8: interleukin-8
IL27-A: interleukin-27 subunit alpha
ITGB1BP2: melusin
KIM-1: kidney injury molecule-1
KLK6: kallikrein-6
LEP: leptin
LOX-1: lectin-like oxidized LDL receptor 1
mAmp: membranebound aminopeptidase P
MB: myoglobin
MCP-1: monocyte chemotactic protein 1
MMP-1: matrix metalloproteinase-1
MMP-10: matrix metalloproteinase-10
MMP-12: matrix metalloproteinase-12
MMP-3: matrix metalloproteinase-3
MMP-7: matrix metalloproteinase-7
MPO: myeloperoxidase
NEMO: NF-kappa-B essential modulator
NTproBNP: N-terminal pro-B-type natriuretic peptide
OPG: osteoprotegerin
PAPPA: pappalysin-1
PAR-1: proteinase-activated receptor 1
PDGF subunit B: platelet-derived growth factor subunit B
PECAM-1: platelet endothelial cell adhesion molecule
PlGF: placenta growth factor
PRL: prolactin
PSGL-1: P-selectin glycoprotein ligand 1
PTX3: pentraxin-related protein PTX3
RAGE: receptor for advanced glycosylation end products
REN: renin
RETN: resistin
SCF: stem cell factor
SELE: E-selectin
SIRT2: SIR2-like protein 2
SPON1: spondin-1
SRC: proto-oncogene tyrosineprotein kinase Src
ST2: ST2 protein
t-PA: tissue-type plasminogen activator
TF: tissue factor
TIE2: angiopoietin-1 receptor
TM: thrombomodulin
TNF-R1: tumor necrosis factor receptor 1
TNF-R2: tumor necrosis factor receptor 2
TNFSF14: tumor necrosis factor ligand superfamily member 14
TRAIL: TNF-related apoptosis-inducing ligand
TRAIL-R2: TNF-related apoptosis-inducing ligand receptor 2
TRANCE: TNF-related activation-induced cytokine
U-PAR: urokinase plasminogen activator surface receptor
VEGF-A: vascular endothelial growth factor A
VEGF-D: vascular endothelial growth factor D
